# 

**Published:** 1890-08-16

**Authors:** 


PRICE ONE PENNY.

203, Vol. VIII.—August 16th, 1890.

[Registered at the General Post Office as a Newspaper for transmission in the United Kingdom and Abroad.]

WITH EXTRA NURSING SUPPLEMENT.

Per Annum, 6s. 6d.; post free.

Monthly Parts, 6d.; post free 8d.

Ditto per Annum, 8s., post free.

### THE HOSPITAL

A WEEKLY JOURNAL OF

